# The Roentgen Ray Treatment of Rodent Ulcer

**Published:** 1903-01-10

**Authors:** W. Kenneth Wills


### HOSPITAL CLINICS AND MEDICAL PROGRESS.

### THE ROENTGEN RAY TREATMENT OF RODENT ULCER

By W. KENNETH WILLS, M.A.Cantab., M.B., B.C.

SINCE Sequeira,[1] in 1901, tried the effect of the Roentgen rays upon cases of rodent ulcer, reports have appeared from many observers of the good results of this treatment. Not many failures in treatment are reported, but the fact that some have occurred make us not unnaturally inquire what percentage of all cases treated are unsuccessful, and why? This question is, of course, impossible to answer now at this early period of the history of X-ray therapeutics. But those who have given it a trial are thoroughly satisfied that they are dealing with a treatment which is in many cases eminently satisfactory. It would be unreasonable to expect perfection in the manipulation at this early time, and it is possible that some of the unsatisfactory cases may be attributed to want of experience and knowledge which will not baffle the therapeutist of the future.

Cases have been reported of pain caused by the rays and of inflammations set up in the vicinity of the trouble, such as in the eye. But it would seem that the balance is in favour of the rays having rather an opposite effect; and as present reported experience goes, such cases are quite exceptional.

Sequeira[2] himself says that in early cases he still regards excision as the best method of treatment, while he also says that the X-rays gave the best result in most instances. So now it is profitable to inquire what position do the X-rays hold in the treatment of rodent ulcer.

It would seem that the quickest method of dealing with small, early cases is excision, wide enough and deep enough; and the quickest result, ensuring permanence, is undoubtedly the best, provided an anæsthetic is no objection, and the scar obtained sightly. Where the trouble is not on the face or exposed parts the cosmetic effect is not a factor. It is claimed for the ray treatment that the resulting scar is sightly and does not lead to contraction and so to distortion of the features. So many cases—a large majority in fact—of Rodent ulcer arise on the face or about the ear that this statement is of great importance; and the effectual treatment which gives rise to the least disfigurement has much to recommend it.

Curetting is so often ineffectual while the scars left are so unsightly that, by itself, this treatment will apparently die out; combined with effectual caustics it still may have a place where better treatment is not available. McFeely[3] has but lately strongly advocated pure formalin used with a local, or general, anæsthetic; but against these forms of treatment the advocate of the X-rays would urge the twofold objection of the severe pain produced, and the need for an anæsthetic.

X-rays have shown their advantage in these respects, viz.: (1) There is no need for anæsthesia; (2) the absence of pain due to treatment, and the relief of such pain as is incident to the affection, which frequently occurs while under the treatment; (3) the resulting scar is sightly, supple, and in most cases shows very little loss of tissue

Anæsthetics are a well-known deterrent to patients who should take surgical advice for cancerous and other affections; the feeble and those for whom anæsthetics are not safe can be subjected to the X-ray treatment without alarm.

Not a few observers have reported that under the X-ray treatment pain ceases.[4] A numb, dead feeling sometimes ensues, and in some cases there is itching, but this latter may be relieved by a simple ointment containing a little cocaine. This fact will outweigh the length of time required for the treatment of some cases.

Another valuable fact which may be placed with this is the decrease of fœtor and discharge noticeable in those under the X-ray treatment. This seems to be only temporary, as the fœtor will return if for some reason the treatment is suspended before much benefit has ensued.

The third consideration is the greatest peculiarity of the X-ray treatment. The rolled edges gradually sink and disappear, while the base rises until it is more flush with the surrounding skin. So that where a large punched-out hole existed, with crater-like edges, a smooth scar remains, leaving an excellent cosmetic result. Some cases reported of this are little short of marvellous.

What then are the objections urged against, and the limitations of, this treatment? It is stated that bone and cartilage are not affected by the X-rays to any appreciable degree, so that where the growth has involved these a complete cure cannot be looked for. The rolled edge takes much longer to disappear than the ulcer takes to fill up; and there may be a danger of producing a dermatitis on the newly formed tissues on endeavouring to remove this edge.

For this the suggestion has been urged that the edges should be removed by the curette before treatment. And undoubtedly this would shorten the process considerably, but in itself is open to the objections named above. However, a judicious admixture of these treatments may be found to give the best results.

The risk of dermatitis: Provided that efficient protection be used for outlying parts, and provided that the treatment is in the hands of one who is conversant with the apparatus and its dangers, this objection is reduced to a minimum.

And this last provision will probably have a bearing upon the great objection of the cost: for those who have frequent use for the apparatus will probably find it easier to work economically. At first there was some discussion as to whether it was necessary to produce a "reaction" or not, and by this is meant a train of phenomena which appear after an exposure to the rays, the duration of which differs according to the individual and according to the tube used. This "reaction" may be anything from a simple erythema to a necrotic action. Most observers agree that a simple reaction must be obtained, while the necrotic "dermatitis" is to be avoided. This erythematous reaction may come on not many hours after the exposure, or it may be delayed for some weeks, when the combined effects of interim exposures lead to a sudden necrotic dermatitis. This explains in itself the need for the treatment to be in skilled hands.

Once obtained the good result does not die down immediately upon the subsidence of the erythema,[5] but seems to consist in a tendency in the cells towards normal growth and a loss of degeneracy.

There is discussion also as to what sort of tube gives the best results, one with a "high" vacuum or a "low" one. It seems that a change of tubes will frequently produce results of a sudden nature whether from a "high" to a "low" or a "low" to a "high." On the whole the expressed opinion seems to be in favour of the "high" tube producing a reaction more easily than the "low." In fact[6] some have gone so far as to assert that it is the electric state in which everthing in the vicinity of a high tube is seen to be which has the actual beneficial effect upon the pathological condition. But this has been denied. Much of this technique has yet to be worked out with certainty, but enough has been published to give the X-rays a place, if not a high place, in the therapeutics of rodent ulcer, among other cutaneous affections. The test of time is still wanting, but even if there should be recurrences, and reports of such cases are recorded in which treatment was not followed up sufficiently long after an apparent cure had been obtained to make that cure more probable, we are in no worse case than the older methods where recurrences were frequent enough.

1 Brit. Med. Jour., Feb. 9, 1901. 2 Brit. Med. Jour., Oct. 25. 1902. 3 Brit. Med. Jour., Nov. 3, 1902. 4 Jour. Amer. Med. Assoc., 1902, xxxviii., p. 911. 5 Brit. Med. Jour., April 12, 1902, 6 Brit. Med. Jour., Oct. 25, 1902.

